# Priority setting: the importance of incorporating opportunity costs

**DOI:** 10.1186/2045-4015-1-45

**Published:** 2012-11-26

**Authors:** Ole Frithjof Norheim

**Affiliations:** 1 Department of Research and Development, Haukeland University Hospital, N-5020, BERGEN, Norway

## Abstract

This article is a comment on Ofra Golan and Paul Hansen’s framework for health technology prioritization that they suggest could be developed and used by the committee selecting new technologies for Israel’s health basket. Although Golan and Hansen should be commended for the innovative way they have started to explore these issues, more work need to be done to take proper account of opportunity costs.

## 

In this issue Ofra Golan and Paul Hansen present a framework for health technology prioritization, and some illustrative cases, that they suggest could be developed and used by the committee selecting new technologies for Israel’s health basket [1]. The framework, they say, is based explicitly on value for money that enables explicit inclusion of information related to health benefits, equity benefits, quality of evidence, health care costs and other “X-factors” that may be of relevance for priority setting among new interventions being considered for priority among essential services. Such a framework is urgently needed, not only in Israel, but also in any system that seek to improve priority setting in an analytic, systematic, and transparent way.

A key motivation for the proposed framework is the need to develop a comprehensive framework that can handle all criteria at the same time, allow for uncertainty or lack of evidence on cost and outcomes, and aggregate all information in a way that is transparent and allows for making trade-offs between different considerations.

For example, they argue that for new technologies there are often lack of precise estimates of clinical benefits or “equity benefits”, so they devised a point-based system assigning weights to broad categories of benefits. For life-prolongation benefits or quality of life improvements, these categories are: 1) None/Very small; 2) Small benefits; 3) Medium benefits; Large benefits. For lives saved the categories are: 1) None; 2) Few: 1–50 lives saved; 3) Some: 51–250 lives saved; 4) Many: 251–500 lives saved; or 5) Very many: > 500 lives saved.

After each technology has been rated on the points system’s dimensions, the values are summed to get a benefit score for each technology assessed. Next, the score is mapped on the y-axis on a ‘Value for Money (VfM) Chart’, where the x-axis shows net incremental total costs to Israel’s health system. In addition, quality of evidence (graded) is indicated by size of ‘bubble’ in the chart, and possible “x-factors” by color symbols. By looking at the chart, the decision makers could use this information as input to further deliberations and subsequent decisions.

Although the underlying motivation and conceptualization is in accordance with many of the ideals of transparent, accountable decision making [2], I am afraid that the framework needs a stronger theoretical foundation. In my view, there already exist methods for aggregating costs [3], health benefits [4], and integrating equity considerations [5] that should not so easily be dismissed.

Consider three examples discussed in the article: traztuzumab (Herceptin) used as adjuvant therapy for some forms of breast cancer; verteporfin (Visudyne) is used to treat a form of macula degeneration (that leads to blindness); and left-ventricular assist devices for severe heart failure. In Norway the permanent committee for quality and priority setting has discussed all three interventions and provided their advice. Left-ventricular assist devices were assessed to cost about 3,4 mill shekel per QALY (analysis not published in English), and is not prioritized in the regular Norwegian health care system. Visudyne is recognized as very costly but somewhat more cost effective (one study showing 1,8 mill to 480 000 shekel per QALY gained [6]) and introduced for some selected indications. Herceptin as adjuvant therapy was found clearly cost effective (at approx. 73 000 shekel per QALY [7]) for selected groups and was quickly prioritized in the Norwegian system.

Golan and Hanson analyze rand conclude, by way of their framework, in exactly the opposite direction. They argue it would be “understandable” if the Basket Committee decided not to fund Herceptin because the total cost are so much higher than for Visudyne and left-ventricular assist devices, and that it is “easy to imagine decision-makers preferring LVAD (… to …) Visudyne”, because it is cheaper.

The reason they reach a different conclusion is that their framework highlights affordability (based on incremental unit cost multiplied with number of patients in need) instead of opportunity costs. This emphasis on affordability is a bit problematic and lacks a clear theoretical foundation. For the cost of treating one patient with LVAD one could treat many patients with breast cancer and gain more than 40 times the QALYs gained from LVAD.

Ethical and political trade-off must be made, but not between health benefits and total costs (as the VfM chart suggests). One may criticize incremental cost-effectiveness analysis, because it in its standard form disregards distributional concerns, but to disregard opportunity costs is probably not the best approach. Even with weak evidence, it is better to aggregate health benefits, as equity weighted QALYs, though modeling, than by a point system based on imprecise categorizations of health benefits. A better alternative could be to build upon Health Technology Assessments, which should include cost-effectiveness analysis; incorporate distributional (and other) concerns after deliberations; and acknowledge strength or lack of evidence explicitly [8].

So my advice to the Basket Committee is this: your criteria for what to fund in the essential basket are sound, but more work is needed before we have a model that can handle all criteria at the same time, allow for uncertainty, and aggregate all information in a way that is transparent and theoretically sound. This said, I want to commend Golan and Hansen for the innovative way they have started to explore these issues. Having such a framework in place would truly enhance the decision making process.

## Competing interests

The author declares that he has no competing interests.

## Authors’ information

Ole Frithjof Norheim is a professor in medical ethics, Dept. of Public Health, University of Bergen and a physician, Haukeland University Hospital. He is currently heading two research projects: Priority setting in global health (2012–2016) and Priority setting across clinical specialties in Norway (2010-2014).

Comment on: Goland and Hansen: “Which health technologies should be funded? A prioritization framework based explicitly on value for money”.
